# Effects of Osteopathic Manipulative Treatment on Rare Diseases: A Case Report on Gordon Syndrome

**DOI:** 10.7759/cureus.71778

**Published:** 2024-10-18

**Authors:** Timothy Johnson, Holly B Waters

**Affiliations:** 1 Osteopathic Medicine, Nova Southeastern University Dr. Kiran C. Patel College of Osteopathic Medicine, Clearwater, USA

**Keywords:** congenital disease, novel, osteopathic manipulative medicine, osteopathic manipulative treatment (omt), osteopathic principles and practice, rare disease

## Abstract

Gordon syndrome (GS), also known as distal arthrogryposis type 3, is a rare congenital disorder characterized by debilitating multisystem defects and for which there is currently no cure. In the absence of a definitive treatment, multimodal symptomatic approaches are employed to enhance quality of life. The case presented involves a 40-year-old female with GS who exhibited multiple chronic, widespread, and severe musculoskeletal ailments that limited her daily functional capacity. This case highlights a novel application of osteopathic manipulative treatment (OMT) aimed at improving the patient’s quality of life by alleviating symptoms associated with GS pathology. A personalized OMT regimen was designed to address her pain and physiological limitations resulting from a variety of somatic dysfunctions. Each appointment included a full-body treatment, with a particular focus on her most severely affected areas. Throughout the course of multiple sessions, both indirect and direct modalities were utilized with notable success. As treatment progressed, the patient reported significant improvements in functionality and reductions in pain across most body regions. While GS is rare, the positive outcomes observed in this case should serve to educate and inspire healthcare providers regarding the diverse OMT techniques available to enhance the quality of life for individuals with GS and others facing significant disease burdens.

## Introduction

Gordon syndrome (GS), or distal arthrogryposis type 3, is an extraordinarily rare congenital disorder inherited in an autosomal dominant manner. Due to its rarity, medical literature has documented GS in only approximately forty individuals from five distinct families [1]. This disorder arises from a mutation in the PIEZO2 gene, which encodes a mechanosensitive ion channel. As a mechanoreceptor, PIEZO2 plays a crucial role in converting sensory stimuli into chemical signals, and it is predominantly found in Merkel cells, where its function is closely related to light touch [2].

GS is characterized by a wide variety of musculoskeletal (MSK) deformities. The most common manifestations include camptodactyly of the fingers and talipes (clubfoot), along with decreased muscle mass and a high palate, all of which typically develop at birth [3]. Similar mutations in the PIEZO2 gene are also found in other genetic disorders, such as distal arthrogryposis type 5 and Marden-Walker syndrome [4-6]. Given its congenital nature, there is currently no standard treatment for GS. Suggested treatment protocols typically involve a multidisciplinary approach tailored to the patient’s functional limitations and specific physical ailments, which may include physical therapy, orthotics or braces, and surgical interventions [1,5].

A review of the existing literature reveals no prior examples of osteopathic manipulative treatment (OMT) being employed in GS patients. Instead, previous efforts have primarily focused on maximizing functionality and minimizing the impact of pathological defects on an individual basis [1,5].

In this case, OMT was utilized as part of a multi-specialty treatment strategy for a woman presenting with numerous MSK complaints stemming from several severe pathologies and associated somatic dysfunctions that significantly limited her quality of life. These pathologies included, among others, amputated bilateral (B/L) talipes accompanied by neuropathies, joint contractures, arthralgias, and myalgias.

## Case presentation

A 40-year-old Caucasian female with GS presented to the OMT clinic on December 21, 2022, with chronic, widespread, severe polyarticular MSK pain (right > left), combined with leva-dextroscoliosis and a mix of flexion and extension contractures due to GS (Table 1). Her primary care physician recommended OMT after she experienced minimal symptomatic relief from trials of physical therapy, chiropractic treatment, dietary modifications, and alternative medicine tinctures. The patient reported that her MSK pain had worsened since birth, and she had undergone multiple surgeries due to her physical deformities. She noted that her arthralgias intensified with cold and wet weather, while her myalgias improved with stretching. Her primary concern was neck stiffness (“inability to hold her head up”) and lower back pain accompanied by B/L sciatica, which interfered with her daily life.

**Table 1:** Osteopathic structural examination findings (12/21/22) B/L, bilateral; ESLRL, extended sidebent left rotated left; ESRRR, extended sidebent right rotated right; Ext., extremity; HTN, hypertonicity; IO, interosseous; L, left; NSLRR, neutral sidebent left rotated right; NSRRL, neutral sidebent right rotated left; OA, occipito-atlantal; R, right

Region	Somatic dysfunction
Head	OA B/L extended
Cervical	C2-C5 ESLRL, C6-7 ESRRR
Thorax	T1-T12 NSLRR with lordosis
Lumbar	L1-L5 NSRRL with exaggerated lordosis
Sacrum	L unilateral extension
Pelvis	R outflare, R posterior rotation
Ribs	L 1-12 inhaled, R 1-12 exhaled
Upper ext.	B/L retracted shoulders, B/L wrists with flexion dysfunction, B/L hands with flattened palmar arches
Lower ext.	R quadriceps HTN, R internally rotated hip, B/L tibia fibula IO membrane restriction, post-surgical changes with multiple surgical stars distally B/L, R piriformis tender point
Abdomen	Significant celiac and superior mesenteric ganglia restrictions, L inhaled hemidiaphragm

The patient’s past medical history included cleft palate, esophageal strictures, hemorrhoids, polyps, diverticulosis, gastroesophageal reflux disease (GERD), and recurrent sinus pain and infections since childhood, with no improvement despite significant lifestyle modifications. She had previously visited an alternative practice provider who administered multiple unknown tinctures, which exacerbated her condition. Additionally, she had a history of with-no-lysine kinase 1 (WNK) and WNK4 mutations affecting her gastrointestinal system.

Her surgical history included B/L foot amputation at age 9 for club feet and recurrent surgical removal of skin tags from the right eardrum. The family history revealed multiple relatives with GS, including her half-brother and nephew, both on her father’s side. Her brother died as a child from an asthma attack, while her nephew was still alive.

The general physical examination revealed that the patient was awake, alert, and oriented to person, place, and condition. The neurologic examination showed diminished but intact sensation in her right arm, with 4/5 grip strength bilaterally and 5/5 strength elsewhere (according to the Medical Research Council Manual Muscle Testing Scale). Cranial nerves II-XII were intact, and deep tendon reflexes were 2+ bilaterally. Her pupils were equal, round, and reactive to light and accommodation, and her cognitive functioning was normal. The MSK examination indicated anterior head carriage, limited extension, and severely restricted B/L rotation of the head. Additionally, she exhibited severe lumbar scoliosis. Although her gait was limited, she could ambulate unassisted with B/L lower extremity prostheses in place.

Our hypothesis was to explore the effectiveness of OMT as a therapeutic approach for pain and diminished functionality in a patient with multiple congenital MSK changes secondary to GS, ultimately aiming to improve her quality of life.

To achieve this goal, treatment and follow-up focused on OMT directed at the patient’s areas of greatest discomfort and disability: the lower legs, lower back, pelvis, abdominal region, and neck. These regions exhibited a multitude of muscular, skeletal, connective tissue, and neuronal somatic dysfunctions, making them prime targets for OMT (Table 1). During her first appointment, we employed multiple modalities, primarily utilizing indirect techniques such as counterstrain, traction articulatory techniques, indirect myofascial release, and soft tissue manipulation, which yielded positive results. Following favorable patient feedback after the first appointment, we implemented a trial of direct treatments. Notably, the patient continued to experience improvement without adverse effects when we incorporated direct techniques, including muscle energy, Still’s technique, lymphatic techniques, direct myofascial release, and compression articulatory techniques, during her second appointment. Significant progress was also noted with muscle energy and Still’s technique applied to articular structures. Additionally, direct myofascial release proved effective in addressing celiac, superior mesenteric, and inferior mesenteric ganglia. All treatments were administered by the authors.

Appointments were scheduled two to four weeks apart at the patient’s convenience. We encouraged her to perform home trapezius and posterior scalene stretches after the first appointment and added a home quadratus lumborum stretch following the second appointment.

At the conclusion of her first treatment, the patient reported significantly reduced neck stiffness and an improved ability to hold her head up. During the first follow-up appointment, she noted decreased symptoms of GERD, reduced back and neck pain (following the resolution of post-treatment myalgias), an increased range of motion, and a considerable enhancement in functionality in her neck. Cervical manipulation during follow-up facilitated full correction of C2-C5 through cervical articulation and Still’s technique, nearly restoring her range of motion. Several weeks after her second appointment, while conversing on the phone, the patient reported significant improvements in her quality of life, attributing these changes to decreased pain and increased range of motion in several body regions (Table 2).

**Table 2:** Patient-completed modified 2003 Cornell Musculoskeletal Discomfort Questionnaire (2/26/23) OMT, osteopathic manipulative treatment

Body region	Before your first OMT appointment, how often did you experience aches, pain, or discomfort in the associated areas? If this has changed since the start of treatment, please indicate your new level.	Before your first OMT appointment, if you experienced pain in that area, please rate it on a scale of 0-10. If the pain changed after any OMT appointment since then, please note the new pain scale 0-10.	Before your first OMT appointment, how did the pain in these areas interfere with your ability to do work? If this has changed since the start of treatment, please indicate the new level.
Neck	Several times every day to one to two times last week	10 to 2	Substantially interfered to Not at all
Shoulder (right/left)	Several times every day to three to four times last week/Never	10 to 2/N/A	Substantially interfered to Slightly interfered/N/A
Upper back	Several times every day	10 to 6	Substantially interfered
Upper arm (right/left)	Never/Never	N/A	N/A
Lower back	Several times every day to once every day	10 to 6	Substantially interfered to Slightly interfered
Forearm (right/left)	Never/Never	N/A	N/A
Wrist (right/left)	One to two times last week/Never	5 to 3/N/A	Slightly interfered/N/A
Hip/buttocks	Several times every day to once every day	10 to 7	Substantially interfered
Thigh (right/left)	Never/Never	N/A	N/A
Knee (right/left)	Three to four times last week/one to two times last week	5 to 2/3 to 0	Slightly interfered/Slightly interfered to Not at all
Lower leg (right/left)	Several times every day to once every day/Never	10 to 5/N/A	Substantially interfered to Slightly interfered/N/A
Foot (right/left)	Several times every day/Several times every day	10 to 10/7 to 2	Substantially interfered/Substantially interfered

## Discussion

It is well documented in the medical literature that OMT can effectively address a wide variety of MSK symptoms associated with both rare and common diseases. Although the effects of OMT on GS are novel, past successes of OMT on various body systems, alongside fundamental physiologic principles, informed the creation of a comprehensive, individualized treatment regimen. Despite the complex etiology of GS and the absence of a definitive treatment, this holistic approach yielded favorable results. If similar outcomes can be replicated in other individuals with GS, it may encourage a broader acceptance of OMT among practicing clinicians, significantly enhancing healthcare access for those living with this condition.

In a stepwise approach, the most problematic areas were targeted first, leading to significant improvement. Drawing from previous successes, diverse MSK manipulations were applied to the neck and shoulders [7,8]. As seen in prior studies, this patient experienced a notable reduction in pain and an increase in functionality in these regions. By addressing both the pain and the reduced functionality resulting from multiple congenital MSK changes associated with GS, we achieved the primary objective of the treatment: improving quality of life. This preliminary MSK intervention had a profound impact on the patient, evidenced by a decrease in both the intensity and frequency of pain, along with enhancements in her ability to perform work and daily activities across nearly every appendage (Table 2).

While the treatment goals centered on reducing pain, increasing functionality, and enhancing quality of life, the patient’s perspective proved to be of greater significance than the provider’s. During the second treatment session, palpation revealed a marked reduction in MSK hypertonicity across multiple body regions. Furthermore, spinal rotation and bony somatic dysfunctions felt subjectively less severe and responded more rapidly to treatment. These physical findings were consistent with the self-reported improvements noted by the patient (Table 2).

A notable secondary complaint was GERD. Drawing from existing research on OMT’s efficacy in treating GERD, similar techniques were applied, resulting in substantial symptomatic relief [9,10]. Techniques employed included celiac and mesenteric ganglia myofascial release, occipito-atlantal decompression, sacral inhibition, and addressing the associated thoracic (T) 5-9 viscerosomatics. Further investigation of the medical literature revealed that the PIEZO2 gene implicated in GS plays a significant role in gastrointestinal peristalsis as well as enterochromaffin cell function [11]. The PIEZO gene, identified in 2010, has already been linked to the pathophysiology of numerous diseases [12]. Continued research into this newly identified, widely distributed gene will be invaluable for future targeted treatments for GS patients. This project further supports the use of OMT in GS patients, demonstrating preliminary success in managing both MSK and gastrointestinal disturbances. Given the positive outcomes observed in the general population and this specific case, it is the author’s opinion that similar beneficial effects of OMT can be expected in other GS patients who frequently experience GERD, inflammatory bowel disease, and irritable bowel syndrome [9-11].

## Conclusions

This case report was limited by the short duration of documented treatment, the absence of objective, measured MSK results, and the lack of a control group. Despite these limitations, the findings suggest that OMT can significantly decrease pain, enhance functionality, and improve quality of life after just two treatment sessions, even in the presence of complex congenital MSK issues associated with GS. The positive outcomes observed in this preliminary study warrant further investigation through a large-scale trial comparing various OMT modalities across different body regions in patients with congenital conditions affecting limb growth. Although GS is rare, the initially successful treatments reported here may inform and improve the management of other patients with similar MSK-related disorders.
